# Impact of Region-of-Interest Delineation on Stability and Reproducibility of Liver SNR Measurements in ^68^ Ga-PSMA PET/CT

**DOI:** 10.1055/s-0043-1768446

**Published:** 2023-05-16

**Authors:** Masoomeh Fooladi, Sahar Rezaei, Farahnaz Aghahosseini, Yalda Salehi, Nima Kasraie, Peyman Sheikhzadeh

**Affiliations:** 1 Department of Medical Physics and Biomedical Engineering, Tehran University of Medical Sciences, Tehran, Iran; 2 Department of Radiology, School of Medicine, Tabriz University of Medical Sciences, Tabriz, Iran; 3 Clinical Research Development Unit, Imam Reza General Hospital, Tabriz University of Medical Sciences, Tabriz, Iran; 4 Department of Nuclear Medicine, Imam Khomeini Hospital Complex, Tehran University of Medical Sciences, Tehran, Iran; 5 Department of Radiology, UT Southwestern Medical Center, Dallas, Texas, United States

**Keywords:** ^68^ Ga-PET, quality control, image quality, signal-to-noise ratio, region of interest

## Abstract

**Objective** This study aims to assess the impact of various regions of interest (ROIs) and volumes of interest (VOIs) delineations on the reproducibility of liver signal-to-noise-ratio (SNRliver) measurements, as well as to find the most reproducible way to estimate it in gallium-68 positron emission tomography ( ^68^ Ga-PET) imaging. We also investigated the SNRliver-weight relationship for these ROIs and VOIs delineations.

**Methods** A cohort of 40 patients (40 males; mean weight: 76.5 kg [58–115 kg]) with prostate cancer were included. ^68^ Ga-PET/CT imaging (mean injected activity: 91.4 MBq [51.2 MBq to 134.1 MBq] was performed on a 5-ring bismuth germanium oxide-based Discovery IQ PET/CT using ordered subset expectation maximization image reconstruction algorithm. Afterward, circular ROIs and spherical VOIs with two different diameters of 30 and 40 mm were drawn on the right lobe of the livers. The performance of the various defined regions was evaluated by the average standardized uptake value (SUV _mean_ ), standard deviation (SD) of the SUV (SUV _SD_ ), SNR _liver_ , and SD of the SNR _liver_ metrics.

**Results** There were no significant differences in SUV _mean_ among the various ROIs and VOIs ( *p* > 0.05). On the other hand, the lower SUV _SD_ was obtained by spherical VOI with diameter of 30 mm. The largest SNR _liver_ was obtained by ROI (30 mm). The SD of SNR _liver_ with ROI (30 mm) was also the largest, while the lowest SD of SNR _liver_ was observed for VOI (40 mm). There is a higher correlation coefficient between the patient-dependent parameter of weight and the image quality parameter of SNRliver for both VOI (30 mm) and VOI (40 mm) compared to the ROIs.

**Conclusion** Our results indicate that SNR _liver_ measurements are affected by the size and shape of the respective ROIs and VOIs. The spherical VOI with a 40 mm diameter leads to more stable and reproducible SNR measurement in the liver.

## Introduction

Gallium-68 prostate-specific membrane antigen positron emission tomography ( ^68^ Ga-PSMA PET) imaging is commonly used in diagnosis, staging, treatment planning, and response monitoring of prostate cancer. 1 2 3 4 5 The standardized uptake value (SUV) of the primary tumor and its local metastases is a PET-derived parameter that can be used to semiquantitatively evaluate radiotracer accumulation. In this regard, it should be noted that quantitative precision and reproducibility of the SUV are very important for both differential diagnosis and monitoring therapy response. Therefore, to achieve accurate quantification during PET scanning, image quality assessment and optimization of PET scanners are necessary. 6 7 A commonly reported standard metric for comparing and evaluating PET scanner performance is based on the use of noise-equivalent count rates (NEC or NECR). 8 9 NEC delivers a good indicator of image quality due to the combination of true, scatter, and random coincidence effects. However, this metric does not take into account the effect of various reconstruction algorithms on image quality. 10

Nowadays, iterative reconstruction algorithms are widely used in PET imaging. However, PET quantification is affected by the reconstruction algorithms. 10 11 12 The signal-to-noise ratio (SNR) in the liver is commonly used as a standard metric for the qualitative and quantitative evaluation of the clinical PET images reconstructed with iterative reconstruction algorithms such as ordered subset expectation maximization (OSEM) and Bayesian penalized-likelihood reconstruction algorithm. 9 13 14

However, SNR in the liver has been shown to have a weak correlation with the visual assessment of PET clinical images. 15 16 On the other hand, liver ^68^ Ga uptake is not high and uniform and the placement of regions of interest (ROIs) or volumes of interest (VOIs) on the liver affects the reproducibility and stability of SNR. 17 Based on PET imaging protocols, circular ROI or spherical VOI with a 30-mm diameter in the right lobe of the liver provides good reproducibility for liver SUV measurement. 18 19 20 In addition, it is well-known that patient-dependent parameter of weight can affect SNR in the liver. Previous studies have shown changes in SNR over a range of patient weights, especially in obese patients with a significant amount of body fat, and despite prescribing weight-based injected activity for them, the accumulation of radiopharmaceuticals and the resulting SNR are still low, which affect the quality of PET images. 21 Therefore, it can be helpful to compare liver SNR for different ROIs and VOIs from the PET acquisition data over a wide range of patient weights.

Given the fact that a stable and reproducible SNR is required in the liver, it is essential to determine the most suitable procedure for ROI or VOI drawing. Importantly, no studies have been performed on the impact of different ROIs and VOIs on liver SNR measurement in ^68^ Ga-PET/CT imaging. Therefore, the main objectives of this study are to evaluate the effect of different ROI and VOI delineations on the reproducibility of liver SNR measurements and also to find the most reproducible way to estimate it in ^68^ Ga-PET images.

## Materials and Methods

### Patients Study

In this study, ^68^ Ga-PET/CT images of forty patients (40 males; mean weight: 76.5 kg [58 kg to 115 kg]; mean body mass index [BMI]: 27.7 kg/m ^2^ [21.8 kg/m ^2^ to 38.9 kg/m ^2^ ]) were recruited through the Imam Khomeini Hospital Nuclear Medicine Centre, in Iran. The mean (range) administered activity of ^68^ Ga-PSMA was 91.4 MBq (51.2 MBq to 134.1 MBq) in accordance with the European Association of Nuclear Medicine guidelines. 22 The whole-body PET/CT scan was acquired 60 minutes post-68Ga-PSMA intravenous injection.

### Data Acquisition and Image Reconstruction

Data acquisition was performed using Discovery IQ PET/CT (GE Healthcare, Milwaukee, Wisconsin, United States). The scanner is comprised of a PET system with 6.3 × 6.3 × 30 mm bismuth germanium oxide detectors, 36 detectors in each ring, an axial field of view of 26 cm, and a 70 cm field of view perpendicular to the axis. All data were reconstructed using OSEM algorithm (VUE point HD + SharpIR) with 4 iterations and 12 subsets. Then, a Gaussian post-smoothing filter of 6.4 mm in full width at half maximum was applied. The plane space of PET images was 3.27 mm. The 16-slice computed tomography (CT) system along with the PET was used for scatter and attenuation correction by 120 kVp and 80 mA.

### Data Analysis

**Fig. 1:**
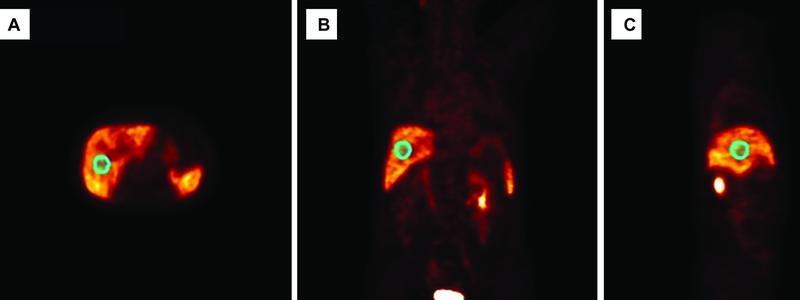
Region-of-interest/volume-of-interest drawings on the liver right lobe: ( **A** ) transverse view, ( **B** ) coronal view, and ( **C** ) sagittal view.

All PET data were analyzed with Amide (Medical Imaging Data Examiner, Los Angeles, United States). According to a similar study by Amakusa et al, 23 circular ROIs with diameters of 30 mm and 40 mm were carefully drawn on the five coronal sequential images of the liver right lobe. For ROI drawing, both the liver hepatic portal and subphrenic areas were not considered. Similarly, spherical VOIs with diameters of 30 and 40 mm were drawn on the liver right lobe ( Fig. 1 ). VOI drawings were repeated five times for each patient. Then, the mean and standard deviation (SD) of SUV _mean_ within the respective ROIs and VOIs were used to compute the SNR for each ROI and VOI as follows:

 For circular ROI and spherical VOI, I = number of ROIs or VOIs for each patient

The following equations along with values of ROIs and VOIs were then used to measure liver SNR: 

For circular ROI and spherical VOI

The SD of liver SNR was also calculated accordingly.

In addition, for each patient, the patient-dependent parameter of weight was collected from the patient files to investigate the relationship between weight parameter and image quality parameter of SNRliver.

### Statistical Analysis

The mean of SUV _mean_ , SUV _SD,_ and SNR _liver_ among different ROIs and VOIs were compared by the one-way analysis of variance, followed by a post hoc least significant difference test with *p* -value less than 0.05 as a significance level. Statistical analysis was performed using SPSS, version 24.0 (IBM Corporation, Chicago, Illinois, United States).

## Results

**Fig. 2:**
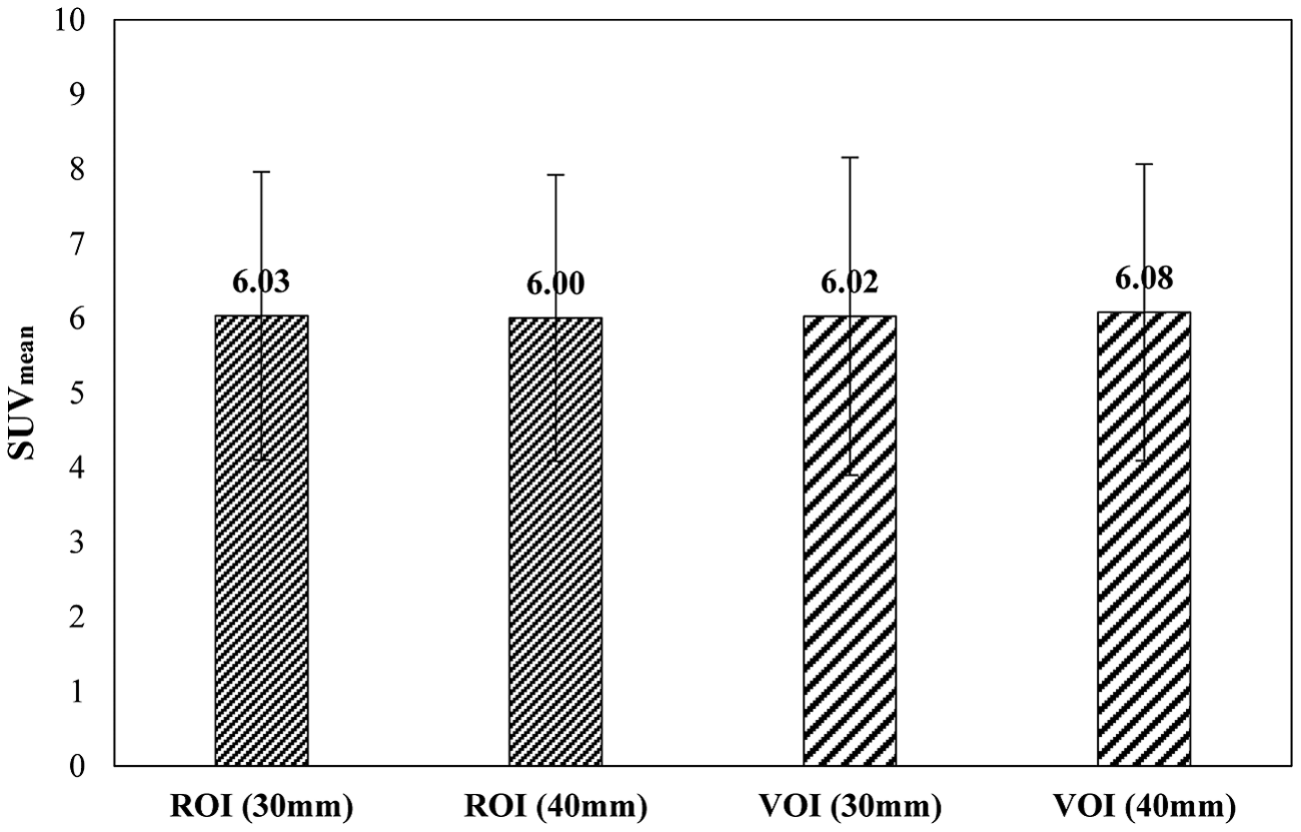
Comparison of the mean standard uptake value (SUV _mean_ ) among various region of interests and volume of interests (ROIs and VOIs). There was no significant difference among them ( *p* > 0.05).

Livers of all 40 patients were analyzed using ^68^ Ga-PSMA PET scan. The comparison of the average SUV _mean_ among the two ROI and two VOI groups is shown in Fig. 2 . The intermethod SUV _mean_ differences for ROI (30 mm) versus ROI (40 mm), ROI (30 mm) versus VOI (30 mm), ROI (30 mm) versus VOI (40 mm), as well as VOI (30 mm) versus VOI (40 mm) were not statistically significant (each *p* > 0.05).

**Fig. 3:**
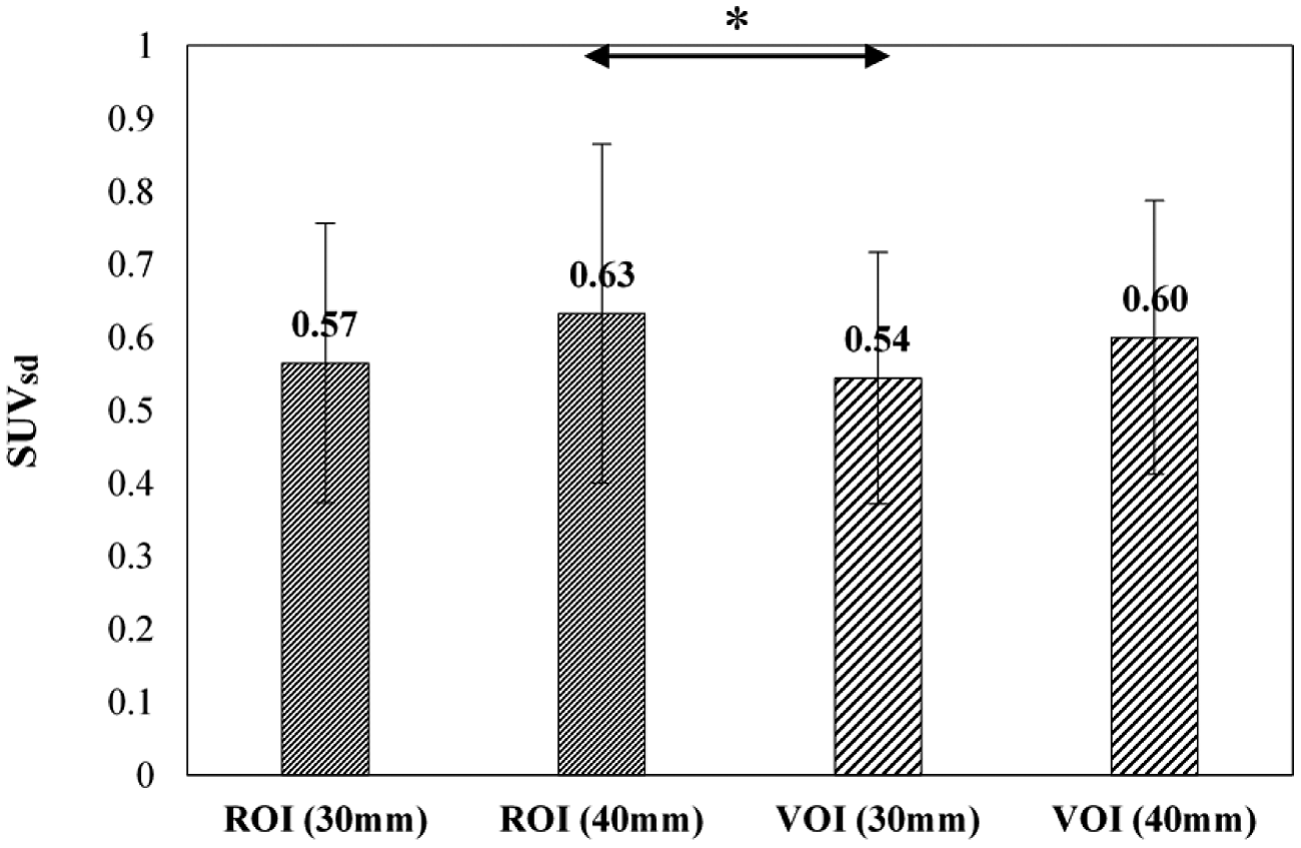
Comparison of the average standard deviation of standard uptake value (SUV _SD_ ) among various region of interests and volume of interests (ROIs and VOIs). The average value of SUV _SD_ in VOI (30 mm) was significantly lower than that of ROI (40 mm) ( *p* < 0.05) but was not significantly lower than that of others. * : < 0.05

Fig. 3 shows a comparison of the average SUV _SD_ among the two ROIs and two VOIs. The VOI (30 mm) provided lower mean SUV _SD_ in comparison with others. There was only a significant difference between SD of ROI (40 mm) and VOI (30 mm) ( *p* < 0.05). Moreover, there was no statistically significant difference in SUV _SD_ among ROI (30 mm), ROI (40 mm), and VOI (40 mm) ( *p* > 0.05).

**Fig. 4:**
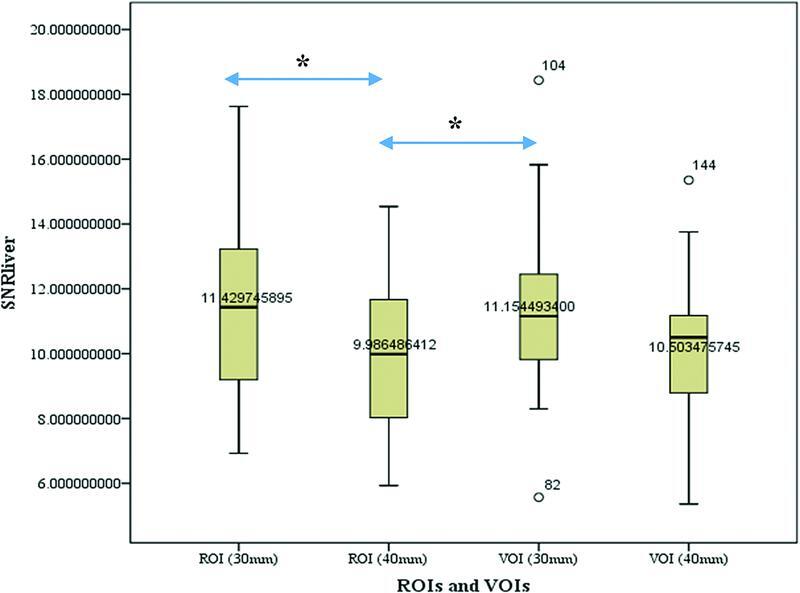
Comparison of the liver signal-to-noise-ratio (SNR _liver_ ) among various region of interests and volume of interests (ROIs and VOIs). There was a statistically significant difference of SNR _liver_ between ROI (30 mm) and ROI (40 mm), as well as ROI (40 mm) and VOI (30 mm) ( *p* < 0.05). * : < 0.05

Fig. 4 shows box plots of SNR _liver_ among various ROIs and VOIs, which calculated based on SUV _mean_ . The intermethod differences of SNR _liver_ values for ROI (30 mm) versus ROI (40 mm) as well as ROI (40 mm) versus VOI (30 mm) were statistically significant (each *p* < 0.05). * : < 0.05

**Fig. 5:**
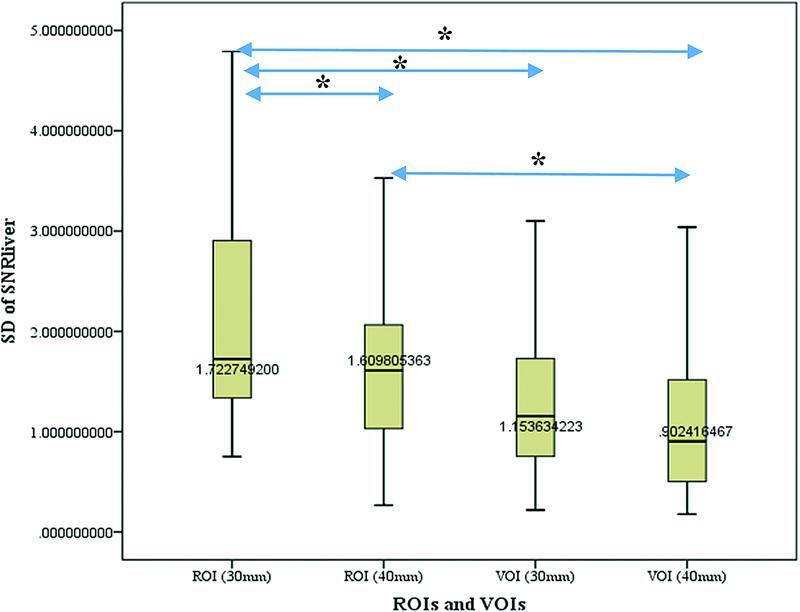
Comparison of standard deviations (SD) of liver signal-to-noise-ratio (SNR _liver_ ) among various region of interests and volume of interests (ROIs and VOIs). The SD of ROI (30 mm) was significantly higher than those of ROI (40 mm), VOI (30 mm) and VOI (40 mm) in descending order ( *p* < 0.05). Also, there was a significant difference between ROI (40 mm) and VOI (40 mm) ( *p* < 0.05). * : < 0.05

Comparison of SDs of SNR _liver_ among two ROIs and two VOIs is also shown in Fig. 5 . The SD difference was significantly higher for ROI (30 mm) compared to others ( *p* < 0.05). There was also a significant difference between SD of ROI (40 mm) and VOI (40 mm) with *p* -value less than 0.05.

**Fig. 6:**
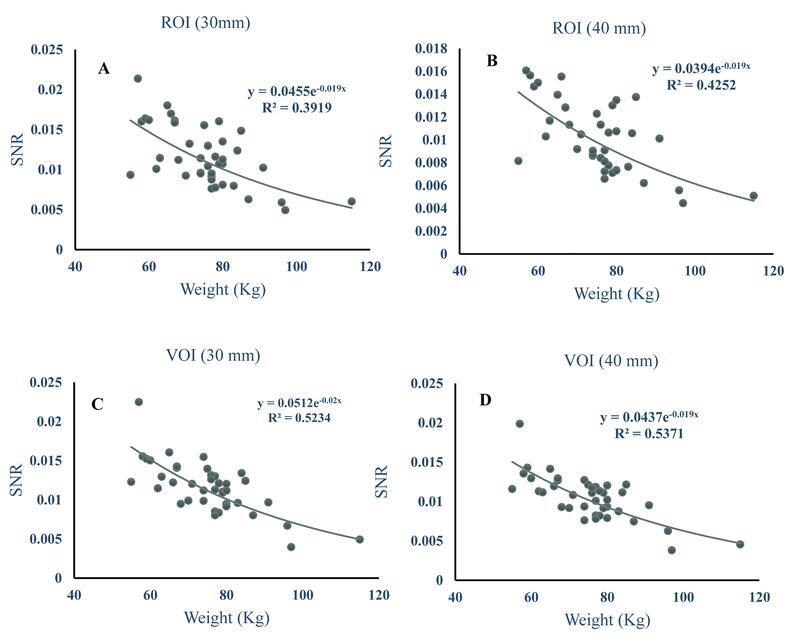
Graphs ( **A–C** ) showing liver signal-to-noise-ratio (SNRs) plotted against patient-dependent parameter of weight. ROI, region of interest; VOI, volume of interest.

There is a higher correlation coefficient between patient-dependent parameter of weight and image quality parameter of SNRliver for both VOI (30 mm) and VOI (40 mm) compared to ROI (30 mm) and ROI (40 mm) ( Fig. 6 ).

## Discussion

Due to the importance of the liver SNR in evaluating image quality, we studied the impact of various ROI and VOI drawings on the stability and reproducibility of liver SNR measurements in ^68^ Ga PET/CT imaging.

Our findings demonstrate that there was no significant difference among ROIs and VOIs in SUV _mean_ ( Fig. 2 ). Therefore, it can be concluded that the selection of the size and shape of the ROI or VOI does not greatly affect the liver SUV mean. Another observation is that the value of SUV _SD_ with VOI (30 mm) was lower compared to other VOI and ROIs, probably due to the drawing area of the VOI ( Fig. 3 ). Moreover, similar to the previous study 23 our findings corroborate that a larger ROI and VOI definition can better represent the overall distribution of ^68^ Ga in the liver.

We also observed that a higher SNR _liver_ value was obtained with ROI (30 mm) ( Fig. 4 ). Although there were no significant differences in SUV _mean_ across all ROIs and VOIs, the observed intermethod variations related to SNR _liver_ may be due to variations in SUV _SD_ .

Furthermore, our results show that the SD of SNR _liver_ decreases with increasing ROIs or VOIs size ( Fig. 5 ). In this regard, the lowest SD of SNR _liver_ was yielded by spherical VOI with a 40 mm diameter versus the corresponding ROI and VOI with a 30 mm diameter. Quantitative analysis of FDG-PET data from sixty patients by McDermott et al 24 showed lower SD in liver SNR measurements by drawing a spherical VOI with a diameter of 50 mm. Thus, we infer that ROI or VOI with a larger diameter can provide more reproducible SNR measurements in the liver. However, in patients with smaller liver, drawing a larger ROI or VOI can be challenging.

The injected dose of ^68^ Ga is one of the key factors in the reproducibility of SNR measurements. Increased coefficient of variation in the liver, followed by a decrease in image quality at lower injection doses, is expected. 25 Therefore, the SD of SNR can be susceptible to variations in ROI or VOI sizes due to the SUV variations (see Figs. 3 and 5 ). On the other hand, BMI can affect the measurements of liver SNR. In this regard, previous studies have shown that higher SUVs can be measured in patients with a higher BMI. 26 27

In this work, the SNR due to the OSEM reconstruction algorithm was evaluated as a function of the patients' weights. The findings have shown a reduced nonlinear fitting. Using other reconstruction algorithms, it is possible to keep the trend line having slope close to zero, which can be further investigated in the future.

^68^ Ga PET imaging has different physical properties than ^18^ F-FDG PET, including lower injected activity, higher positron mean energy and hence longer mean free path length, as well as the ability to emit secondary gamma photons with high energy. According to the mentioned characteristics, ^68^ Ga provides a higher SNR in the liver than ^18^ F, which affects image quality. 28 29 In the study conducted by Amakusa et al, 23 the effect of ROI determination on SNR measurement in the liver on ^18^ F-FDG PET images was investigated. Therefore, it seems necessary to conduct a similar study on ^68^ Ga PET images.

The present work has some limitations. All patients were analyzed regardless of BMI classification and signal to background ratio, particularly in obese patients. Further research is necessary for evaluating the liver SNR in different liver sizes and patients with heterogeneous liver. Furthermore, although ROIs and VOIs were evaluated with two sizes, this study only investigated circular ROIs and spherical VOIs.

## Conclusion

Our work highlights the dependence of liver SNR on ROIs or VOIs size and shape. Our results provide evidence that a spherical VOI with a diameter of 40 mm leads to more stable and reproducible SNR measurements in the liver. Overall, in liver SNR measurements, the size and shape for ROIs and VOIs need to be selected carefully.
